# Cross-Correlation between Biosecurity Measures and the Detection of Viral and Bacterial Agents on German Farms with Respiratory Disease

**DOI:** 10.1155/2024/6205899

**Published:** 2024-08-20

**Authors:** Julia Stadler, Kathrin Lillie-Jaschniski, Sophia Zwickl, Susanne Zoels, Sebastiaan Theuns, Mathias Ritzmann, Nick Vereecke

**Affiliations:** ^1^ Clinic for Swine at the Centre for Clinical Veterinary Medicine Ludwig-Maximilians-University München, München, Germany; ^2^ CEVA Tiergesundheit, Düsseldorf, Germany; ^3^ PathoSense BV, Lier, Belgium; ^4^ Laboratory of Virology Faculty of Veterinary Medicine Ghent University, Merelbeke, Belgium

## Abstract

Effective porcine health management relies majorly on diagnostic tests, vaccination, treatment strategies, and a proper biosecurity management plan. However, understanding the link between circulating microbes and biosecurity measures on a pig farm is not evident. Substantial progress has been made in recent years with the availability of new diagnostic tools (e.g., sequencing-based diagnostics) and extensive biosecurity management questionnaires. However, the interpretation and correlation of these results are hampered by the abundance of gained (meta)data. Therefore, we aimed to cross-correlate viral and bacterial pathogens with respiratory tropism detected by third-generation nanopore metagenomic sequencing with biosecurity measures assessed by Biocheck.UGent™. The study was conducted on 25 sow farms with attached nurseries in Germany with known respiratory distress. The biosecurity level of the study farms complied with the European averages. Interestingly, the farms with the highest biosecurity score showed the lowest overall prevalence of porcine reproductive and respiratory syndrome virus (PRRSV) and *Actinobacillus* sp.; the circulation of well-studied pathogenic viruses, such as PRRSV, was correlated with overall lower biosecurity scores, a higher number of stillborn piglets, and cocirculation of porcine parvovirus type 7. Moreover, potential risk factors for lesser-known agents (e.g., porcine hemagglutinating and encephalomyelitis virus, porcine respiratory coronavirus, and porcine polyomavirus) could also be addressed. For the bacterial pathogen *Glaesserella* sp., a correlation with increased clinical signs was observed, whereas *Lactobacillus* sp. and *Moraxella* sp. are putative biomarkers for pig farms with better biosecurity scores. In conclusion, in-depth cross-correlation of (meta)data from new diagnostic platforms with biosecurity measures on pig farms may contribute to a better understanding of new actions in adapting biosecurity measures. This will not only contribute to improved animal welfare and economic productivity but also helping to address (new) zoonotic disease threats and potential treatments.

## 1. Introduction

Prevention of infectious diseases on swine farms has not only implications on animal welfare and economic productivity, but it is also important in the context of One Health to tackle potential hazards and threats originating from zoonotic diseases [1, 2, 3]. Successful prevention of pathogens requires a multilevel approach, including a minimum of reliable diagnostic tests, vaccination, and treatment strategies, as well as a proper biosecurity management plan. The implementation of new diagnostic tests, including the use of new sequencing approaches (e.g., Oxford Nanopore Technologies) [4, 5, 6, 7], represents a real revolution in our ability to study and understand (co-)circulation of various viral and bacterial pathogens [4, 5, 6, 7]. The introduction of these technologies in the field of (veterinary) diagnostics has enabled a more holistic diagnostic approach to understand the complex interaction between viral and bacterial pathogens involved in respiratory diseases [6, 8]. Moreover, these techniques also allow the (co-)identification of new and emerging pathogens that might pose a threat to pigs and humans [9].

While the identification of circulating pathogens is important for a veterinarian to install treatments and or vaccinations, biosecurity management resembles another important pillar of disease prevention and management. Therefore, continuous verification of biosecurity measures is of importance to minimize the risk of the introduction and spread of pathogens within and between farms [10]. Various studies have assessed risk factors associated with porcine reproductive and respiratory syndrome virus (PRRSV), swine influenza A virus (swIAV), or *Mesomycoplasma hyopneumoniae* (formerly known as *Mycoplasma hyopneumoniae*) [11, 12, 13, 14, 15, 16, 17, 18, 19]. However, little is known about risk factors contributing to the occurrence of so far lesser-studied agents like porcine parainfluenza virus (PPIV; also known as porcine respirovirus or respirovirus suis based on the 2022 release of the ICTV taxonomy), porcine respiratory coronavirus (PRCV), and porcine pneumovirus (PneumoV). The availability and broader implementation of informative biosecurity questionnaires (e.g., Biocheck.UGent™ (BioCheck)) [20] has led to an increased amount of (meta)data on the farm level in various countries (https://biocheckgent.com/). This platform was shown of high value in porcine [21], poultry [20], and cattle [22] management and has been exploited to study the importance of biosecurity management in productivity, performance, antimicrobial usage, and infectious diseases [20, 21, 22, 23]. The latter study only represents a literature review without actual correlations between pathogen detection and biosecurity measures. To the author's knowledge, no studies have directly linked this extended (meta)data with the detection of known and lesser-studied viral and bacterial pathogens across multiple farms in a country. Therefore, the present study aimed to evaluate the cross-correlation between pathogens with respiratory tropism detected by third-generation nanopore metagenomic sequencing and biosecurity parameters from 25 German farms with respiratory distress.

## 2. Materials and Methods

### 2.1. Study Design and Sampling Procedures

The metagenomics and swIAV incidence data used in the current manuscript were previously published in Vereecke et al. [8]. In short, a cross-sectional study was conducted on 25 prospectively selected sow farms with attached nursery units throughout Germany between March 2021 and February 2022. In all farms, frequent respiratory distress was reported by the farmer and the herd attending veterinarian. In addition, all farms had a history of swIAV based on previous detection by RT-qPCR and/or hemagglutination inhibition test. For further swIAV confirmation and for differential diagnosis, sampling of nasal and tracheobronchial swabs (NS and TBS, respectively) was performed as described previously [24, 25]. Four different age groups were sampled, including the start (4–6 weeks of age), mid (7–8 weeks of age), and end (9–10 weeks of age) of the nursery for all farms. From farm eight onward, NS and TBS from four suckling piglets (2–3 weeks of age) originating from four different litters of gilts were also collected. The swIAV incidence at sampling was verified using an RT-qPCR on pools of NS (five animals) and pools of TBS (five and four animals for nursery and suckling piglets, respectively). The RT-qPCR was performed using a modified generic Matrix (M)-gene-specific influenza A virus protocol, according to Spackman [26]. The study design was reviewed by the Ethic Commission of the Ludwig-Maximilians University (LMU) Munich (accession number 254-10-02-2021).

### 2.2. Metagenomic Long-Read Viral and Bacterial Profiling

**Figure 1:**
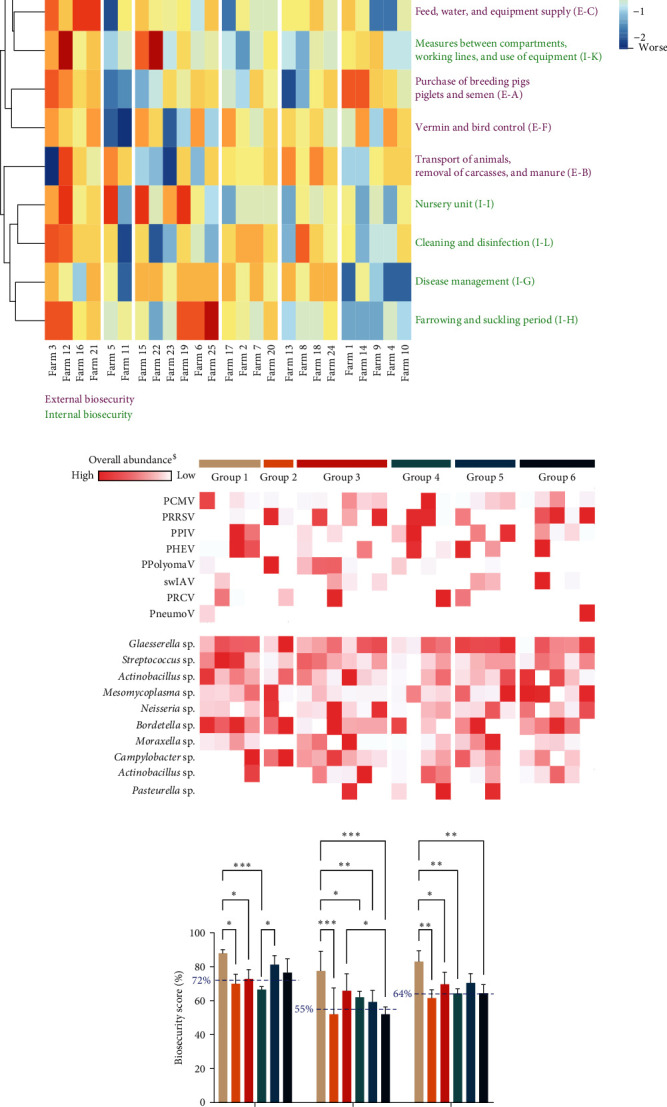
Summary and clustering of all 25 German farms based on their external (purple) and internal (green) biosecurity (BioCheck) scores. (a) Clustered heatmap of all 25 German farms highlighting the Euclidian distances between each farm, with the identification of six biosecurity groups (*x*-axis) and Biocheck external and internal biosecurity categories (*y*-axis). The color-code within the heatmap represents better/higher (red) and worse/lower biosecurity scores as represented by their respective Euclidian distances; (b) prevalence (%) of overall nanopore-based microbial detection of selected relevant viruses and bacteria as highlighted in red (high) and white (low); ^$^exact values of overall abundances are represented in detail in *Supplementary table 1* (gray table); (c) comparative analysis of external (purple), internal (green), and total (black) biosecurity scores (%) for each of the identified groups and significant differences in scores between these groups ( ^*∗*^: *p* < 0.05,  ^*∗∗*^: *p* < 0.01,  ^*∗∗∗*^: *p* < 0.001).

The metagenomic profiles of viral and bacterial populations within each TBS pool were performed at the PathoSense laboratory (Merelbeke, Belgium). For each age group, a freshly pooled sample was processed through an *in-house* developed sample-collection-to-diagnostic-interpretation workflow, applying Oxford Nanopore Technologies' long-read platform, as described before [5, 6, 27]. A detailed overview of the workflow has been described in Vereecke et al. [8]. Read files were deposited in European Nucleotide Archives (ENA) under Project accession PRJEB59352 (ERS14550157-ERS14550248). For association purposes, overall farm incidence was considered rather than age-group-specific incidence. Thus, the relative abundances of each viral and/or bacterial agent were summarized per farm by taking the highest relative abundance (%) over all age groups. These results were summarized in Figure 1(a) and *Supplementary table 1*.

### 2.3. Collection of Metadata on Clinical Signs, Biosecurity, Farm Characteristics, Performance, and Vaccination on Farms

Clinical signs were assessed using a coughing and sneezing index [28], described in detail in Vereecke et al. [8]. To assess biosecurity levels, Biocheck.UGent™ (BioCheck) was performed on each farm (https://biocheckgent.com/en) [21]. In short, a questionnaire of 96 questions allowed to assess both external (purchase of breeding pigs, piglets, and semen (E-A), transport of animals, removal of carcasses, and manure (E-B), feed, water, and equipment supply (E-C), visitors and farmworkers (E-D), vermin and bird control (E-E), and location of the farm (E-F)) and internal (disease management (I-G), farrowing and suckling period (I-H), nursery unit (I-I), finishing unit (I-J), measures between compartments, working lines, and use of equipment (I-K), and cleaning and disinfection (I-L)) biosecurity quantification on each farm. Of note, the internal biosecurity subclassification finishing unit (I-J) was not evaluated in our study because the focus of our trial was on piglet production until the end of the nursery period. In addition, information on farm characteristics (number of sows, number of nursery pigs, number of boars, number of people working on the farm, years of experience in keeping pigs, oldest building on the farm, and newest building on the farm, batch interval, length of suckling period, replacement rate (%), performance parameters (return to estrus (%), abortion rate (%), number of life born piglets, number of stillborn piglets, number of weaned piglets, farrowings per sow per year, and number of piglets lost till weaning), and vaccination state (vaccinations against Erysipelas and swIAV for sows, *Actinobacillus pleuropneumoniae*, *Mesomycoplasma hyopneumoniae*, PRRSV, and porcine circovirus type 2 (PCV2) for piglets and sows) were collected for each farm. The obtained (meta)data were summarized in *Supplementary table 1*.

### 2.4. Multidimensional Biosecurity Clustering and Ranked Cross-Correlation Analyses

Due to the multidimensionality of the Biocheck (meta)data parameters, sublevel biosecurity scores of all farms were compared and summarized, allowing a biosecurity-based clustering of all studied farms as represented by a clustered heatmap. This was done for all farms using the pheatmap package (v1.0.12; https://cran.r-project.org/web/packages/pheatmap/index.html) with the following settings: scale = “row,” clustering_distance_rows = “euclidean,” clustering_distance_cols = “euclidean,” clustering_method = “complete,” cutree_cols = 4. Statistical significance between different Biocheck scores was assessed using a two-way ANOVA, followed by a *post hoc* Tukey's multiple comparisons test as determined in GraphPad Prism (v.10.0.2). Significance was reported when adjusted *p*-values ≤ 0.05 were observed. To evaluate the cross-correlation between viral/bacterial prevalence (as obtained from metagenomics), clinical signs, internal/external biosecurity measures, and other available (meta)data, ranked cross-correlation analyses were performed. Here, the corr_cross function within the lares R package (v5.2.1; https://github.com/laresbernardo/lares) was used. This function allows to evaluate the ranked cross-correlation across all variables, including 69 microbial variables and 179 (meta)data variables, and was run using the following settings: rm.na = T, max_pvalue = 0.05, grid = T, type = 1, method = “spearman,” max = 0.99, pvalue = T. Only cross-correlations with a *p*-value ≤ 0.05 were retained. The resulting Spearman correlation coefficient (SCC) was interpreted as follows: perfect (1), very strong (≥0.7), moderate correlation (0.45–0.7), fair and weak/poor correlation (<0.45) [29]. For simplicity, the latter were not considered in the current manuscript. Complete outputs can be found in *Supplementary table 2*.

## 3. Results and Discussion

### 3.1. Overall Biosecurity on Studied German Farms

With the increasing interest in One Health-focused research, the field of veterinary medicine has been subjected to great changes over the last few years. This is exemplified by the significant efforts that have been made on the reduction of the use of various antimicrobial drugs, which led to an overall reduction of 58.9% since the start of the actions in Germany in 2011 (https://www.bmel.de) [30]. Still, One Health represents a broader concept with a focus on the human–animal-ecosystem, including understanding the (co-)circulation of pathogens and their potential zoonotic risk, animal welfare, animal productivity, and biosecurity [31]. Most of these aspects have been and are currently studied in more depth due to the availability of new technologies. The use of nanopore sequencing for diagnostics is an example of such an emerging tool for diagnostic purposes. It allows for the simultaneous identification of viral and bacterial pathogens in a sample, represented in a semi-quantitative way [4, 5, 6, 7]. Also, from the perspective of biosecurity, the availability of informative questionnaires, such as Biocheck, allows to understand and picture potential breaches in biosecurity on a given farm [20]. With the increasing availability of data associated with new omics approaches, it is important for researchers to mine all available (meta)data to understand and unravel potential links between qualitative and quantitative aspects of their data. Hence, this study focused on cross-correlating viral and bacterial pathogens with over 100 metadata parameters on 25 German farms, suspicious of respiratory disease. The BioCheck analyses included a questionnaire containing 96 questions, which allowed us to determine a total biosecurity score, which can be further divided into an overall internal and external biosecurity score. These again can be subdivided into specific biosecurity subcategories, with each evaluated by their own unique questions within the questionnaire. As shown in Figure 1(a), six and five biosecurity categories represent the overall external (purple) and internal (green) biosecurity scores, respectively. A multidimensional clustering analysis allowed us to cluster our 25 farms into six groups, based on their biosecurity scores at each category. Also, overall abundances of circulating viral and bacterial agents are linked to each of the farms in the heatmap (Figure 1(b)). Among these six groups, one group (group 1 in beige) showed significantly higher external, internal, and total biosecurity scores as compared to most of the other groups (Figure 1(c)). This group, comprising four farms (4/25), showed a mean external (88% ± 2%), internal (78% ± 12%), and total (83% ± 6%) biosecurity score, which is higher than the German BioCheck averages of 72%, 55%, and 64% for external, internal, and total biosecurity score, respectively. Interestingly, the lowest overall prevalence of PRRSV and *Actinobacillus* sp. was detected in this group. These same observations were found for farm group 5 and group 2 for PRRSV and *Actinobacillus* sp., respectively (Figure 1(b)). Indeed, unlike group 2, group 5 had the second-best external and total biosecurity scores (Figure 1(c)). Of note, group 2 only comprised two farms and should not be considered with highly significant power for that reason. The other groups showed variable scores for the three biosecurity measures without impactful significance amongst the groups. Also, their mean biosecurity scores corresponded mostly to the German BioCheck average scores. Overall, the included farms performed at least as good as the predefined German biosecurity averages on internal, external, and total biosecurity scores. As summarized in *Supplementary table 1*, the German farms from this study tend to have low scores (52% ± 20%) on the external biosecurity subcategories feed, water, and equipment supply (E-C), which, however, complies with other European pig farms (53% ± 12%) and with the worldwide measure of 49% (*Supplementary table 3*). For the internal measures, farrowing and suckling period (59% ± 18%; I-H) and measures between compartments, working lines, and use of equipment (50% ± 18%; I-K) the German farms included in our study showed lowered scores compared to the worldwide average of 64% (I-H) and 57% (I-K). However, the scores of our farms were in line with the European averages of 56% ± 10% (I-H) and 53% ± 6% (I-K) for these subcategories (*Supplementary tables 1 and 3*). Thus, in line with the European tendency, a lack of compliance in specific external (feed, water, and equipment supply) and internal biosecurity practices (farrowing and suckling period, measures between compartments, working lines, and use of equipment) was evident in the study farms. Interestingly, the subcategory “measures between compartments and the use of equipment (I-K)” was also identified as one of the most critical points in previous studies [21, 23, 32, 33]. This highlights the need for raising awareness on those biosecurity aspects. A small fraction (16%) of the farms showed significantly better biosecurity measures for all three assessed levels. Interestingly, in those farms PRRSV and *Actinobacillus* sp. were less frequently detected, thus illustrating that implementation of biosecurity practices can potentially reduce the probability of introduction and the spread of pathogens. Thus, to better understand and link the relationship between biosecurity practices and the circulation of various relevant viral and bacterial respiratory pathogens, a ranked cross-correlation analysis was performed on all available data (i.e., 69 microbial and 179 biosecurity and other variables) from the 25 German farms (see *Supplementary table 1*).

### 3.2. Viral Correlations with Biosecurity Measures

For the ranked cross-correlation analyses, only correlations with a significance of *p* < 0.05 were retained for the identified virus (*n* = 23). To maintain focus, only viruses with known or suggestive respiratory tropism were highlighted further. A complete overview of the cross-correlation analysis can be accessed in *Supplementary table 2*. As described before, porcine cytomegalovirus (PCytomegaloV), PRRSV, porcine parainfluenza virus (PPIV), porcine hemagglutinating and encephalomyelitis virus (PHEV), porcine polyomavirus (PpolyomaV), swIAV, PRCV, and porcine pneumovirus (PneumoV) were identified in 19 (76%), 14 (56%), 12 (48%), 10 (40%), 9 (36%), 8 (32%), 6 (24%), and 2 (8%) of the 25 farms, respectively [8]. Of note, this type of analysis relies solely on the statistical power of cross-correlations between the given input parameters. Hence, our available biosecurity and pathogen parameters, as collected on the 25 German farms, serve as a first dataset to assess the added value of cross-correlating microbial detection (*i.e*., using metagenomic long-read sequencing) with biosecurity measures (*i.e*., BioCheck). Due to the intrinsic high variability in this type of dataset, increased numbers of farms from a single country, across Europe, or even worldwide would further improve the statistical power of the cross-correlations as presented in the current study. In this perspective, it is important to note that cross-correlation of highly dimensional datasets might result in the identification of random significant events. This again could be prevented by the inclusion of more replicates to increase statistical power or more complex statistical approaches. For that reason, the significant cross-correlations reported in the current manuscript were classified to be strong or moderate correlations based on their SCC. The latter implies the potential underestimation of biosecurity measures to be correlated across multiple viral/bacterial species. Also, the rapid development of more thorough and accurate diagnostic and biosecurity management tools will increase the usefulness of our cross-correlation approach.

**Figure 2:**
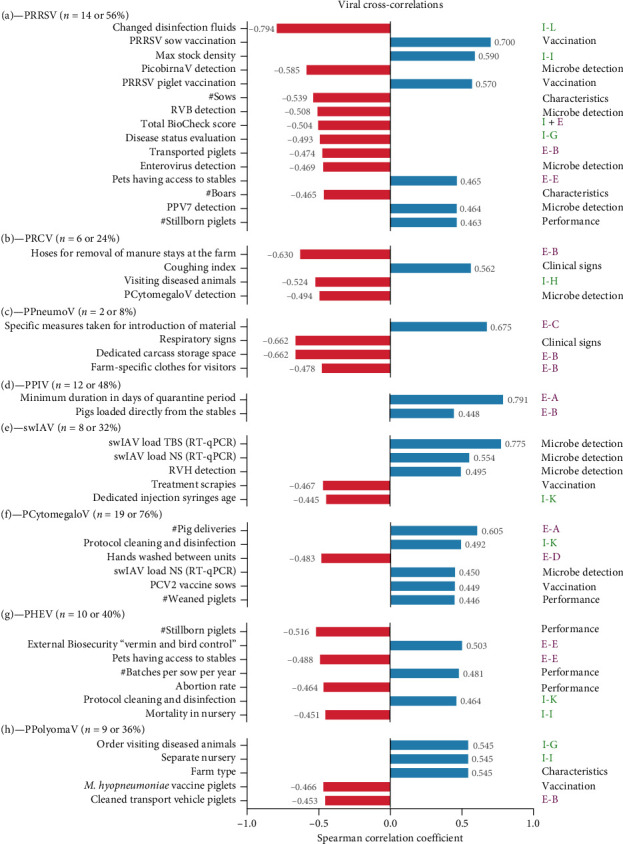
Ranked cross-correlations of selected viruses, highlighting positive (blue) and negative (red) Spearman correlation coefficients of significant (*p* < 0.05) correlations. Ranked cross-correlations for porcine reproductive and respiratory syndrome virus (PRRSV) (a), porcine respiratory COVID-19 (PRCV) (b), porcine pneumovirus (PPneumoV) (c), porcine parainfluenza virus (PPIV) (d), swine influenza A virus (swIAV) (e), porcine cytomegalovirus (PCytomegaloV) (f), porcine hemagglutinating and encephalomyelitis virus (PHEV) (g), and porcine polyomavirus (PPolyomaV) (h) are shown. *Abbreviations*. RT-qPCR = reverse transcription qPCR; # = number; PCV2 = porcine circovirus type 2; TBS = tracheobronchial swab; NS = nose swab.

In general, our observations divided our selected microbes into those showing correlations likely supporting their pathogenic or dubious (*i.e*., commensal or facultative) nature. This can be exemplified by the direct link between PRRSV and a lowered overall BioCheck score, as well as a higher number of stillborn piglets (0.46 SCC) (Figure 2(a)). Indeed, our best-scoring farms (group 1) also showed the lowest overall prevalence of PRRSV (Figure 1(a)). In addition, our data showed a high cross-correlation with farms where disinfection fluids of boot baths are not regularly changed (−0.79 SCC; I-L). Comparably, maximal stocking densities (0.59 SCC; I-I), absence of frequent disease evaluation (−0.49 SCC; I-G), and pets having access to stables (0.47 SCC; E-E) also represent potential biosecurity breaches for higher PRRSV pressure. These findings are in line with previous studies determining the risk factors for PRRSV detection [14, 16] and should be considered as important parameters for biosecurity management on farms with the persistence of PRRSV. Of note, sow and piglet PRRSV vaccination also showed a correlation (0.70 SCC and 0.57 SCC, respectively), which is not surprising due to the broad use of live-attenuated vaccination strategy, resulting in the detection of PRRSV during the metagenomic screening in studied samples [34]. The suggested cross-correlation with porcine parvovirus type 7 (PPV7) detection (0.46 SCC) is an interesting finding, as previous studies showed high levels of PPV7 in serum, which might impact the severity of PRRSV infection [35]. Still, the dependency of porcine parvoviruses to replicate in actively dividing immune cells also does not rule out its potential opportunistic character as its cross-correlation might be closely linked to a previous viral (*e.g*., PRRSV), bacterial, or multimicrobial infection [27].

Comparably, the suggestive clinical character of PRCV was supported by the observed correlation with a higher coughing index (0.56 SCC; Figure 2(b)). PRCV leads to disruption of the mucociliary apparatus and decreases the function of pulmonary alveolar macrophages, which reduces the ability of macrophages to clear bacteria. This eventually results in clinical signs such as dyspnea, tachypnea, sneezing, coughing, anorexia, and reduced growth performance. Interestingly, sharing of hoses for manure removal between farms (−0.63 SCC; E-B) and no separate management of diseased and healthy pigs (−0.52 SCC; I-H) were identified as potential risk factors for the circulation of PRCV under the condition of our study. On the contrary, the pathogenicity of PPneumoV (Figure 2(c)) and PPIV (Figure 2(d)) are not much elaborated; only a few publications try to understand the role and spread of these pathogens in swine herds [8, 36, 37, 38, 39, 40, 41]. Results of the present study indicate that the absence of a designated and demarcated loading area for pigs might serve as risk factors for the presence of PPIV (0.45 SCC; E-B). In addition, PPneumoV was more frequently detected in farms with the absence of dedicated carcass storage space (−0.66 SCC; E-B) and the absence of farm-specific cloths for visitors (−0.48 SCC; E-B). For both pathogens no cross-correlation with clinical signs could be noticed under the condition of our study. Controversially, PPneumoV was even more present in farms without clinical signs (*i.e*., sneezing and coughing) (−0.66 SCC). For both PRCV (Figure 2(b)) and PPneumoV (Figure 2(c)), a low prevalence (24% and 8%, respectively) was shown across the 25 farms in our study. Hence, care should be taken when concluding on cross-correlation with these small datasets. Also, this must be interpreted with caution, as clinical signs were only assessed once, and PPneumoV/PPIV might also have, just as other respiratory viruses, a predisposing role as part of the respiratory disease complex. It has been shown for swIAV that the presence of the virus does not necessarily correlate with the occurrence of clinical signs, as those often result from secondary bacterial infections promoted by the previous viral infection [42]. For swIAV, multiple methodologies were applied to assess the presence of swIAV in both NS and TBS from different age groups. The NS samples were investigated for swIAV by RT-qPCR. For the TBS samples, both the RT-qPCR and metagenomic sequencing protocols were performed on pooled samples. Including data from these different experimental sources in the cross-correlation analysis resulted in a very strong correlation between the swIAV genome copy equivalents obtained from the RT-qPCR and metagenomic sequencing of the TBS samples (0.78 SCC; Figure 2(e)). For the NS samples, a moderate positive correlation (0.55 SCC) was observed with the metagenomic detection of swIAV in pooled TBS samples. Interestingly, the detection of swIAV could be linked with farms that do not use dedicated injection syringes specific for each age group (−0.45 SCC; I-K). Also, Lopez-Moreno et al. [43] demonstrated that tools used for piglet handling can facilitate the transmission of swIAV. In addition, the correlation between swIAV and the absence of scrapies treatment (−0.47 SCC) might suggest the potential of insects as mechanical vectors. Transmission of influenza virus through ectoparasites has been documented for birds [44, 45, 46], but so far, information on the actual role of this route of transmission in pigs is missing.

Only a few studies exist investigating the role of PCytomegaloV in respiratory diseases. In accordance with Martín-Valls et al. [47], a correlation between PCytomegaloV and swIAV could be observed in our investigations (i.e., detection of swIAV in NS on RT-qPCR (0.45 SCC); Figure 2(f)). In addition, our study suggests that a reduction of the frequency of pig deliveries (0.61 SCC; E-A) and washing and disinfection of hands might reduce the risk of PCytomegaloV (−0.48 SCC; E-D). However, the correlation between PCytomegaloV and the number of weaned piglets likely supports its dubious nature (0.45 SCC). The dubious nature of some viruses in the study was also exemplified by PHEV (Figure 2(g)), which was correlated with less stillborn piglets (−0.52 SCC), lower abortion rates (−0.46 SCC), low mortality in the nursery (−0.45 SCC; I-I), better external biosecurity scores for vermin and bird control (0.50 SCC; E-E) and with farm where pets had no access to the stables (−0.49 SCC; E-E), and where protocols for cleaning and disinfection were implemented (0.46 SCC; I-K). Indeed, its suggestive pathogenic role in respiratory disease has been studied to some extent, showing that PHEV can infect naïve pigs of any age, but clinical disease is variable and age-dependent. Also, possible differences in virulence of viral variants have been described [48]. The virus is widespread, as most seroprevalence studies performed worldwide showed a high prevalence (e.g., 96% of sow farms tested in the United States) [49]. Additionally, it was shown that PHEV circulated sub-clinically in these herds. Acute disease mainly occurs in piglets under 3–4 weeks of age, born from naïve sows, with mortality rates reaching 100%. These animals show either clinical signs of vomiting and wasting disease and/or neurological signs, including tremors, recumbency, padding opisthotonos, and finally, death. Although PHEV can replicate in the respiratory epithelium, the role of PHEV as a respiratory pathogen remains dubious and awaits confirmation by further investigation [50, 51]. The same is true for the role of PPolyomaV (Figure 2(h)), for which our investigation also suggests a dubious nature (*i.e*., cross-correlations with farms having the nursery separated from sows (0.55 SCC; I–I), farms where diseased animals were constantly visited after the healthy ones (0.55 SCC; I-G), farms without cleaning and disinfection of transport vehicle (−0.45 SCC; E-B), farms not using *M. hyopneumoniae* vaccination for piglets (−0.47 SCC)).

### 3.3. Bacterial Correlations with Biosecurity Measures

The same cross-correlation analyses were performed for bacterial microbes. Again, only correlations with a significance of *p* < 0.05 were retained for the identified bacteria (*n* = 36). To maintain focus, bacteria with known or suggestive respiratory tropism are further highlighted. A complete overview of the cross-correlation analysis can be accessed in *Supplementary table 2*. As described before, bacteria belonging to the *Glaesserella*, *Streptococcus*, *Lactobacillus*, *Mesomycoplasma*, *Neisseria*, *Bordetella*, *Moraxella*, *Campylobacter*, *Actinobacillus*, and *Pasteurella* were identified in 25 (100%), 24 (96%), 24 (96%), 23 (92%), 21 (84%), 19 (76%), 18 (72%), 18 (72%), 16 (64%), and 4 (16%) of the 25 farms [8]. It is important to note here that our metagenomic data were obtained with the R9.4.1 nanopore chemistry, which has a median raw read accuracy of 95%–97%. This represents an important limitation of the current study, as only bacterial classifications at the genus-level were considered. The implementation of the new R10.4.1 (also known for its +Q20 chemistry or >99% raw read accuracy) nanopore flow cell, which is available at the time of writing and its duplex read-out, is thought to enable the classification of bacteria at the species level in future projects [52, 53]. Hence, the results of the bacterial cross-correlations must be interpreted cautiously due to this genus-level classification, as they might compromise both known pathogenic and dubious/facultative/commensal species, as exemplified by *Mesomycoplasma hyopneumoniae* and *Mesomycoplasma hyorhinis* [54]. Thus, a more generalized interpretation of the (meta-)data will be presented here.

**Figure 3:**
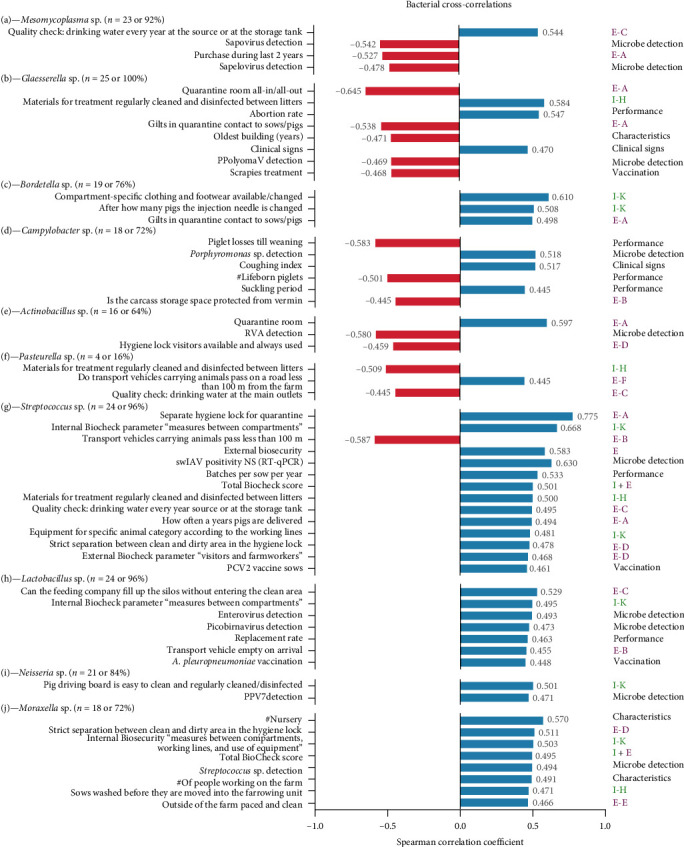
Ranked cross-correlations of selected bacteria, highlighting positive (blue) and negative (red) Spearman correlation coefficients of significant (*p* < 0.05) correlations. Ranked cross-correlations for *Mesomycoplasma* sp. (a), *Glaesserella* sp. (b), *Bordetella* sp. (c), *Campylobacter* sp. (d), *Actinobacillus* sp. (e), *Pasteurella* sp. (f), *Streptococcus* sp. (g), *Lactobacillus* sp. (h), *Neisseria* sp. (i), and *Moraxella* sp. (j). *Abbreviations*. RT-qPCR = reverse transcription qPCR; # = number; PCV2 = porcine circovirus type 2; TBS = tracheobronchial swab; NS = nose swab.

Indeed, our analyses only showed cross-correlations of *Mesomycoplasma* sp. with farms on which a quality check for the drinking water is in place (0.54 SCC; E-C; Figure 3(a)). Hence, it is important to mention that *Mesomycoplasma* sp. and *Glaesserella* sp. were identified on 92% of all farms, which might impact the cross-correlation. Nevertheless, our cross-correlations were performed on overall relative abundances and not solely on detection (present/absent), which adds an additional quantitative dimension to our cross-correlation analyses. Increasing the number of farms, countries, and health statuses would favor current and future analyses in perspective of obtaining more statistic power. Unfortunately, the use of newly emerging technologies, such as Oxford Nanopore Technologies, currently represents a limitation in the number of analyzed samples due to the current cost per sample. Nevertheless, this rapidly evolving field is thought to result in a further decrease in sequencing costs in the near future, allowing bigger sample sizes at a lower cost [55]. Although our cross-correlations suggested the pathogenic nature of *Glaesserella* sp. as it was correlated with the absence of an all-in/all-out quarantine room (−0.65 SCC; E-A), higher abortion rates (0.55 SCC), worse clinical signs (0.47 SCC), and absence of scrapies treatment (−0.47 SCC; Figure 3(b)), those results should be interpreted cautiously due to the detection of *Glaesserella* sp. in 100% of the farms. Moreover, the metadata on quarantine room all-in/all-out only was only available from 11 out of 25 farms (*Supplementary table 1*). Comparable observations were made for *Bordetella* sp. (Figure 3(c), 76%); correlated with the number of pigs injected with a single needle (ranging from 1 to 5 L) (0.51 SCC; I-K) and gilts having contact with sows/pigs (0.50 SCC; E-A), *Campylobacter* sp. (Figure 3(d), 72%); correlated with higher coughing index (0.52 SCC), less life born piglets (−0.50 SCC) and carcass storage spaces not protected from vermin (−0.45 SCC; E-B), *Actinobacillus* sp. (Figure 3(e), 64%); correlated with hygiene lock for visitors not available or not always used (−0.46 SCC; E-D) and the presence of a quarantine room (0.60 SCC; E-A), and *Pasteurella* sp. (Figure 3(f), 16%); correlated with treatment materials not regularly cleaned (−0.51 SCC; I-H), transport vehicles with animals passing nearby (<100 m) (0.45 SCC; E-F) and no quality check of the drinking water (−0.45 SCC; E-C).

Surprisingly, *Streptococcus* sp. (Figure 3(g), 96%) was more present in farms with a higher total external biosecurity score (0.58 SCC; E), internal biosecurity measure “measures between compartments” (0.67 SCC; I-K), and external biosecurity measure “visitors and farmworkers” (0.47 SCC; E-D), farms with a strict separation between clean and dirty areas in the hygiene lock (0.48 SCC; E-D), where equipment for specific animal categories are used according to the working lines (0.48 SCC; I-K), and with a separate hygiene lock for quarantine (0.78 SCC; E-A). On the contrary, cross-correlations were observed for more frequent pig deliveries per year (0.49 SCC; E-A), having a higher number of batches per sow per year (0.53 SCC) and on which swIAV was detected in NS using RT-qPCR (0.63 SCC). This is another example on which the genus-level analysis is thought to represent an even bigger bias on *Streptococcus* sp. cross-correlations [56, 57, 58]. Hence again, caution should be taken when drawing conclusions, particularly from our cross-correlation of *Streptococcus* sp., which could include *S. dysgalactiae* and *S. porcinus* species among others, as they cannot be distinguished from *Streptococcus suis* strains [59]. Moreover, no clear factors have been identified to differentiate between pathogenic and commensal *S. suis* strains. Thus, the exact role and pathogenicity of *S. suis* within the porcine respiratory disease complex has long been debated [60]. Of note, the strain-level differentiation will also remain a problem when applying the R10.4.1 chemistry as it will not allow strain-level (*i.e*., pathogenic versus commensal) discrimination either. Our analyses also revealed evidence of a dubious/facultative/commensal nature for *Lactobacillus* sp. (96%; Figure 3(h)), *Neisseria* sp. (84%; Figure 3(i)), and *Moraxella* sp. (72%; Figure 3(j)). Thereby, both *Lactobacillus* sp. and *Moraxella* sp. were even positively correlated with the overall biosecurity score or sublevel biosecurity scores (*e.g*., internal biosecurity measure “measures between compartments, working lines, and use of equipment” (0.50 SCC; I-K) and (0.50 SCC; I-K), respectively) and might as such be considered potential commensal/beneficial organisms. Furthermore, *Lactobacillus* sp. was cross-correlated with vaccination against *A. pleuropneumoniae* (0.45 SCC) and on farms on which the feeding company is able to fill up the silos without entering the clean area (0.53 SCC; E-C). *Moraxella* sp. has been detected frequently in the respiratory tract, but the exact role needs further clarification [61, 62]. It was also cross-correlated with other measures of high biosecurity, such as farms where the outside is paced and clean (0.47 SCC; E-E), farms on which sows were mostly (sometimes or always) washed before they were moved into the farrowing unit (0.47 SCC; I-H), and farms with strict separation between clean and dirty area in the hygiene lock (0.51 SCC; E-D). More importantly, these results suggest that linking microbial and biosecurity (meta)data is also able to highlight the potential identification of microbial biomarkers for which its presence can be linked to increased (overall) biosecurity levels.

When summarizing our cross-correlations (*Supplementary table 4*), the location of the farm (E-F) seemed not to be correlated with any of the studied microbes. This is in contrast to previous studies [63, 64, 65] and might be explained by the fact that more than half of the study farms were located in areas with higher proximity between farms (16/25 farms had no other pig farms within a 500-m radius). Interestingly, the purchase of breeding pigs, piglets, and semen (E-A) showed more cross-correlations with bacteria (*n* = 7) as compared to viruses (*n* = 2). Aversely, the transport of animals, removal of carcasses, and manure (E-B) showed more correlations with viruses (*n* = 6) than with bacteria (*n* = 3). In addition, the external subcategories E-C (feed, water, and equipment supply) and E-D (visitors and farmworkers) were correlated more with bacteria (*n* = 4) and less with viruses (*n* = 1). When looking into internal biosecurity subcategories, viral diseases were correlated with all different subcategories. However, bacteria were only correlated with the farrowing and suckling period (I-H; *n* = 4) and measures between compartments, working lines, and use of equipment (I-K; *n* = 7). All other additional (*i.e*., non-BioCheck questionnaire) parameters were evenly correlated between viral and bacterial microbes.

## 4. Conclusions

With the current study, we hope to help explore and share all (meta-)data to understand and decipher the impact of biosecurity (or other) measures on the spread of viral and bacterial pathogens. Although our study produced several interesting results, our limitations (i.e., sample size, bacterial genus level, randomness of significance when comparing highly dimensional data, and statistical power of the ranked cross-correlation analyses) should be highlighted again. Nevertheless, the authors believe that this type of analysis, as presented in our results, highlights important trends in biosecurity measures that could be implemented and investigated to improve overall biosecurity in German farms. Even though current literature on risk factor assessment focuses majorly on well-studied and prevalent pathogens (e.g., PRRSV, swIAV, and *Mesomycoplasma hyopneumoniae*), this work identified some potential risk factors for so far lesser-studied agents, such as PRCV and PPneumoV. Expanding our knowledge of pathogen complexes through emerging technologies (e.g., sequencing-based diagnostics) and biosecurity tools (e.g., BioCheck questionnaires) and their linkage will represent a revolution in our understanding of pathogen transmission. Finally, we believe that the advent of artificial intelligence will have an important impact in the future on the analysis/linking/association of the increasingly available (meta-)data in pig health management. Overall, this may contribute to a better understanding of new actions in adapting biosecurity measures to improve animal welfare and economic productivity, as well as to help address (new) zoonotic disease threats and potential treatments.

## Data Availability

All datasets used and described in the current manuscript have been made available within the manuscript or its supplementary material. Also, raw read files were deposited in European Nucleotide Archives (ENA) under Project accession PRJEB59352 (ERS14550157-ERS14550248).

## Supplementary Materials

Supplementary 1 Table 1: metadata on clinical signs, biosecurity measures, farm characteristics, performance parameters, and vaccination status of all 25 farms combined with relative abundances of each viral and/or bacterial agent.

Supplementary 2 Table 2: complete overview of cross-correlations between viral/bacterial prevalence (as obtained from metagenomics), clinical signs, internal/external biosecurity measures, and other available (meta)data.

Supplementary 3 Table 3: external, internal, and overall biosecurity scores of different countries in Europe and worldwide.

Supplementary 4 Table 4: summary of cross-correlation between virus/bacteria and internal/external biosecurity measures and other available (meta)data.
